# Astragaloside IV Ameliorates Airway Inflammation in an Established Murine Model of Asthma by Inhibiting the mTORC1 Signaling Pathway

**DOI:** 10.1155/2017/4037086

**Published:** 2017-10-25

**Authors:** Hualiang Jin, Limin Wang, Bei Li, Cui Cai, Jian Ye, Junbo Xia, Shenglin Ma

**Affiliations:** ^1^Department of Respiratory Diseases, Hangzhou First People's Hospital, The Fourth Clinical College of Zhejiang Chinese Medical University, Hangzhou, Zhejiang 310006, China; ^2^Department of Respiratory Diseases, Hangzhou First People's Hospital, Nanjing Medical University, Hangzhou, Zhejiang 310006, China; ^3^Department of Geriatric Medicine, Hangzhou First People's Hospital, Nanjing Medical University, Hangzhou, Zhejiang 310006, China; ^4^Department of Geriatric Medicine, Hangzhou Red Cross Hospital, Hangzhou, Zhejiang 310002, China; ^5^Department of Oncology, Hangzhou First People's Hospital, The Fourth Clinical College of Zhejiang Chinese Medical University, Hangzhou, Zhejiang 310006, China

## Abstract

Astragaloside IV (AS-IV), a main active constituent of* Astragalus membranaceus*, has been confirmed to have antiasthmatic effects. However, it remained unclear whether the beneficial effects of AS-IV on asthma were attributed to the mTOR inhibition; this issue was the focus of the present work. BALB/c mice were sensitized and challenged with ovalbumin followed with 3 weeks of rest/recovery and then reexposure to ovalbumin. AS-IV was administrated during the time of rest and reexposure. The characteristic features of allergic asthma, including airway hyperreactivity, histopathology, cytokines (IL-4, IL-5, IL-13, IL-17, and INF-*γ*), and CD4^+^CD25^+^Foxp3^+^Treg cells in bronchoalveolar lavage fluid (BALF), and downstream proteins of mTORC1/2 signaling were examined. AS-IV markedly suppressed airway hyperresponsiveness and reduced IL-4, IL-5, and IL-17 levels and increased INF-*γ* levels in the BALF. Histological studies showed that AS-IV markedly decreased inflammatory infiltration in the lung tissues. Notably, AS-IV inhibited mTORC1 activity, whereas it had limited effects on mTORC2, as assessed by phosphorylation of mTORC1 and mTORC2 substrates S6 ribosomal protein, p70 S6 Kinase, and Akt, respectively. CD4^+^CD25^+^Foxp3^+^Treg cells in BALF were not significantly changed by AS-IV. Together, these results suggest that the antiasthmatic effects of AS-IV were at least partially from inhibiting the mTORC1 signaling pathway.

## 1. Introduction

Allergic asthma is a chronic inflammatory lung disease of the airway that is typically characterized by airway hyperresponsiveness (AHR), inflammation, mucus hypersecretion, and increases in Th2 cytokines [1]. Despite the widespread use of glucocorticoids and bronchodilators in the past few decades, the incidence and prevalence of asthma remain high [2]. And most patients develop asthma symptoms or exacerbations when they are reexposed to seasonal allergens or stimuli [3]. Therefore, it is imperative that the mechanisms underlying this disease be identified and new therapies be developed for improving disease management and reducing acute asthma attacks. mTOR signaling has been demonstrated to play an important role in T cell proliferation and differentiation [4]. In recent years, studies showed that inhibition of mTOR could attenuate key characteristics of allergic asthma, including airway inflammation, AHR, and goblet cell metaplasia [5]. Rapamycin is a potent mTOR inhibitor. However, studies have indicated the adverse effects of rapamycin, such as body weight loss, increased risks of infection and cancer, and diabetes-like symptoms [6, 7]. Therefore, it is clinically significant to screen new drugs that target mTOR signaling with mild adverse effects.

Astragaloside IV (AS-IV) is the main active constituent of* Astragalus membranaceus*, a Chinese herb that is traditionally used to prevent asthma attacks in chronic asthma patients. Recently, AS-IV was confirmed to have a broad range of pharmacological properties, including immunomodulatory, anti-inflammatory, antifibrosis, antineoplastic, and antidiabetic properties [8]. In addition, AS-IV has been demonstrated to alleviate airway inflammation, AHR, and remodeling in models of asthma when administered early in the disease process [9]. Nevertheless, relevant data of effects of AS-IV during allergen reexposure or established allergic disease remain scarce. And the mechanisms through which AS-IV exerts its antiasthmatic effects have not been fully elucidated. Previously, AS-IV was shown to effectively inhibit mTORC1 activity in different cell lines and mouse models [10]. Therefore, the goal of this study was to confirm antiasthmatic effects of AS-IV in a model of established asthma as well as to investigate the possible mechanisms of AS-IV action, especially with respect to the mTOR signaling pathway.

## 2. Materials and Methods

### 2.1. Materials and Chemicals

Astragaloside IV (purity > 98%) was purchased from Ronghe Co. (Shanghai, China). OVA (purity > 98%, Grade V) and methacholine (purity > 98%) were purchased from Sigma Chemical Co. (St. Louis, MO, USA). Imject alum adjuvant was obtained from Thermo Fisher Scientific Co. (Waltham, MA, USA). Dexamethasone sodium phosphate injection (purity > 99%; 5 mg/mL) was provided by Tianjin pharmaceutical group Xinzheng Co. (Tianjin, China). Rapamycin (purity > 98%) was purchased from LC Laboratories (Woburn, MA, USA). ELISA kits for INF-*γ*, IL-4, IL-5, IL-13, and IL-17A, FITC-labeled anti-rat CD4, APC-labeled anti-rat CD25, and PE-labeled anti-rat Foxp3 were purchased from eBioscience Co. (San Diego, USA). Antibodies for phospho-S6 ribosomal protein (Ser235/236) #4858, phospho-Akt (Ser473) #4060, Akt (#4691), phospho-4E-BP1 (Ser65) #9451, phospho-p70 S6 Kinase (Thr389) #9234, and *β*-actin were obtained from Cell Signaling (Danvers, MA, USA).

### 2.2. Animals

Female pathogen-free BALB/c mice, weighing 20–22 g, were purchased from Shanghai SLAC Co. (Shanghai, China). Seventy mice were randomly divided into seven groups of 10 mice each as follows: a saline control group, an OVA reexposed group, a dexamethasone group, a rapamycin group, and three AS-IV (10, 20, or 40.0 mg/kg) groups. Animals were housed in stainless steel cages in a temperature controlled environment (22 ± 2°C) on a 12 h light-dark schedule (lights on from 6:00 am to 6:00 pm) and given free access to water and rodent chow. The animals were acclimatized for at least 7 days before use in experiments. The animal study protocols were approved by the Institutional Animal Care and Use Committee of Zhejiang Chinese Medical University (permit number: 11-0092; November 20, 2015).

### 2.3. Allergic Sensitization and Challenge

**Figure 1:**
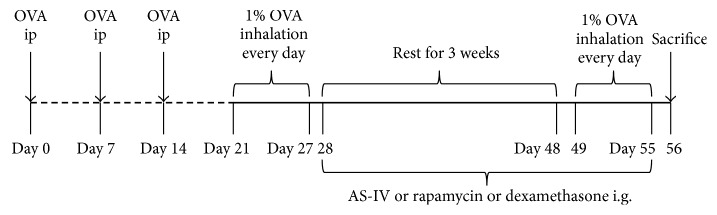
Experimental protocol. Mice were immunized 3 times with intraperitoneal injection of a suspension containing 40 *μ*g OVA (Grade V) and 0.05 mL alum adjuvant. One week after the immunization, mice were challenged by exposure to an aerosol of 1% OVA for 1 week, which was followed by 3 weeks of rest. Then, mice were reexposed to OVA challenge for 1 week. From days 28 to 55, mice in the treatment groups were given oral AS-IV at 10, 20, or 40.0 mg/kg; 1 mg/kg/day dexamethasone (Dex); or 4 mg/kg/day rapamycin (Rapa).

As shown in Figure 1, a murine model of asthma was developed by OVA sensitization and inhalation. Briefly, mice were immunized at 0, 7, and 14 days by a peritoneal injection of 0.2 mL of sterile saline containing 40 *μ*g of OVA and 0.05 mL of alum adjuvant. One week after sensitization, the mice were challenged with aerosolized OVA for 30 min/day each day for one week, which was followed by 3 weeks of rest. Then, mice were reexposed to OVA challenge for 1 week. Meanwhile, rapamycin (4 mg/kg), dexamethasone (1 mg/kg), AS-IV (10, 20, or 40.0 mg/kg), or vehicle (0.25% PEG400, 0.25% Tween 20 in dH2O) was given by i.g. administration one day and then continued for 4 weeks during the rest and OVA reexposure time.

### 2.4. Measurement of AHR

The technique for measuring noninvasive respiratory function was previously described [11]. The pulmonary function was measured by whole-body plethysmography (Buxco*ʼ*s unrestrained WBP systems). WBP application involved measuring “box flow,” which was the net (or sum) of nasal and thoracic flows. Actual flows were calculated in the software considering the temperature, humidity, and pressure. After a stable baseline was achieved, mice were given aerosolized PBS or various concentrations of methacholine (6.25, 12.5, 25, or 50 mg/mL) via a jet nebulizer in the chamber. The enhanced pause (Penh) index of airway hyperreactivity was used as an indicator of changes in airway resistance.

### 2.5. Preparation and Analysis of BALF

Immediately following the assessment of AHR, BALF was obtained by inserting a tracheal tube and lavaging the lung two times with 0.8 mL of sterilized normal saline containing 2% bovine serum albumin. The collected lavage fluid was centrifuged at 500 ×g at 4°C for 10 min. The supernatants were harvested and stored at −80°C for cytokine production measurements. The pellets were resuspended in 1 mL of PBS for flow cytometric analysis. The interleukin (INF-*γ*, IL-4, IL-5, IL-13, and IL-17A) levels in the BALF supernatant were analyzed by ELISA according to the manufacturer's instructions.

### 2.6. Histological Analysis

After BALF was isolated, lung tissue slices were inflated with 10% neutral-buffered formalin. Thin sections (3-4 *μ*m) were cut from blocks and stained with hematoxylin-eosin. Image-Pro Plus software was used for histopathological analysis. The severity of inflammatory cell infiltration in the lung was evaluated with a 5-point scoring system as follows: 0, no cells; 1, a few cells; 2, a ring of cells 1 cell layer deep; 3, a ring of cells 2–4 cells deep; 4, a ring of cells 4-5 cells deep; and 5, a ring of cells > 5 cells deep.

### 2.7. Flow Cytometric Analysis

Cells from the BALF were analyzed for CD4^+^CD25^+^Foxp3^+^ expression using a Treg cell staining reagent according to the manufacturer's instructions. Briefly, prepared cells were washed by centrifugation in flow cytometry staining buffer. Then, cells were stained with FITC-labeled anti-CD4 and APC-labeled anti-CD25 antibodies in staining buffer for 30 min at 4°C. Next, cells were fixed and permeabilized in a fixation/permeabilization solution for 30 min and subsequently stained with 0.5 *μ*g of anti-rat Foxp3 PE. Finally, cells were resuspended in flow cytometry staining buffer and analyzed by flow cytometry using a FACSCalibur instrument with CellQuest software (BD Biosciences, Mountain View).

### 2.8. Western Blot Analysis

Western blot analysis was performed on lung homogenates using the following antibodies: phospho-S6 ribosomal protein Ser235/236 (P-S6RP, 1 : 1000), phospho-4E-BP1 Ser65 (P-4E-BP1, 1 : 1000), phospho-p70 S6 Kinase Thr389 (P-p70S6K, 1 : 1000), Phospho-Akt Ser473 (P-Akt, 1 : 1000), and Akt levels (1 : 1000). To control for protein loading, P-S6RP, P-4E-BP1, and P-p70S6K were normalized to the *β*-actin level, and P-Akt was normalized to the total Akt levels. The HRP-conjugated secondary antibodies were goat anti-mouse and goat anti-rabbit (1 : 5,000). The proteins were detected by using an ImageQuant LAS 4000 ECL System.

### 2.9. Statistical Analysis

Prism 6 software (GraphPad Software, San Diego, CA) was used to perform statistical analysis. The data are expressed as the means ± standard error of mean (SEM). The significance of the differences between groups was determined by one-way ANOVA, followed by post hoc Dunnett's tests. For comparison of the two groups, Student's *t*-test was used. Differences with *P* values < 0.05 were considered statistically significant.

## 3. Results

### 3.1. AS-IV Attenuated AHR to Methacholine

**Figure 2:**
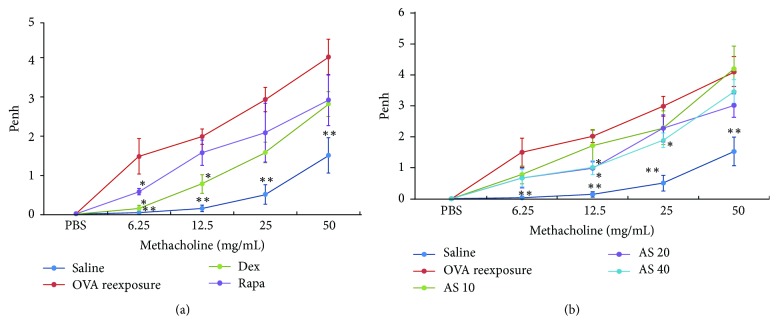
Airway responsiveness to aerosolized methacholine was evaluated by Buxco's whole-body barometric plethysmography in awake, unrestrained mice. Group size: *n* = 10. The mice were nebulized with PBS followed by increasing doses (6.25 to 50 mg/mL) of methacholine. The enhanced pause (Penh) index of airway hyperreactivity was used as an indicator of changes in airway resistance. (a) The AHR was significantly increased after OVA reexposure compared to saline controls. Rapamycin (Rapa) (4 mg/kg/day) and dexamethasone (Dex) (1 mg/kg/day) suppressed the OVA-induced increases in the AHR after allergen reexposure. (b) AS-IV at 20 or 40.0 mg/kg had a remarkably inhibitory effect on the AHR. The data are expressed as the mean ± SEM. ^*∗*^*P* < 0.05 and ^*∗∗*^*P* < 0.01 versus OVA reexposure.

To evaluate the effect of AS-IV on OVA-induced AHR, the airway responsiveness to aerosolized PBS or methacholine was assessed within 24 h after the final challenge. The enhanced pause (Penh) index was measured as an indicator of bronchial responsiveness to inhaled methacholine. Only mild changes in Penh were observed in saline control mice; however, upon allergen reexposure, the AHR significantly increased (Figure 2(a)). Dexamethasone and rapamycin both had inhibitory effects on the reduction in the AHR provoked by methacholine at 6.25 or 12.5 mg/mL (Figure 2(a)). At a methacholine dose of 12.5 mg/mL, oral administration of 20.0 and 40.0 mg/kg AS-IV significantly improved the AHR (Figure 2(b)), and at methacholine dose of 25 mg/mL, AS-IV at 40.0 mg/kg remarkably reduced the AHR (Figure 2(b)).

### 3.2. AS-IV Reduces Inflammatory Cytokine Levels in BALF

**Figure 3:**
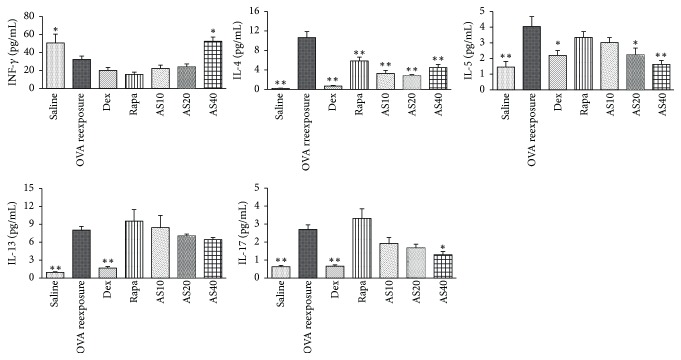
The effect of AS-IV on cytokine secretion in BALF in a murine model of asthma. Secreted levels of the INF-*γ*, IL-4, IL-5, IL-13, and IL-17 cytokines were measured by ELISA. The data are expressed as the mean ± SEM. ^*∗*^*P* < 0.05 and ^*∗∗*^*P* < 0.01 versus the OVA reexposure group.

Airway inflammation in asthma is characterized by an imbalance of Th1/Th2 cytokines as well as elevated Th17 inflammation. Therefore, to determine whether AS-IV could modulate this imbalance, the INF-*γ*, IL-4, IL-5, IL-13, and IL-17A levels were measured. As shown in Figure 3, OVA reexposed mice had significantly elevated levels of IL-4, IL-5, IL-13, and IL-17A and reduced INF-*γ* levels in BALF compared to saline mice (Figure 3). By contrast, oral administration of AS-IV significantly inhibited the upregulation of IL-4, IL-5, and IL-17A, while it increased Th1 cytokine INF-*γ* in the BALF (Figure 3). Dexamethasone significantly inhibited cytokines of IL-4, IL-5, IL-13, and IL-17A, whereas rapamycin only reduced the IL-4 level (Figure 3). However, unlike AS-IV, neither dexamethasone nor rapamycin had a significant effect on the INF-*γ* levels.

### 3.3. AS-IV Inhibited Infiltration of Inflammatory Cells into the Lung

**Figure 4:**
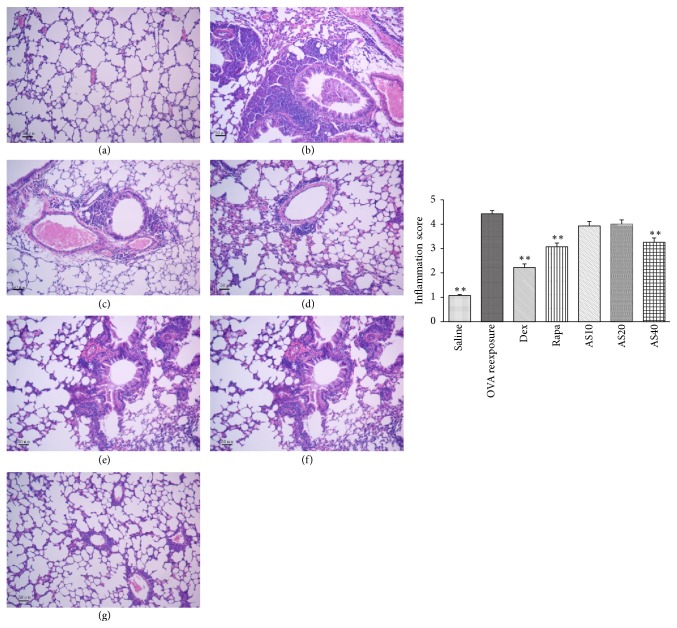
The effect of AS-IV on airway inflammation in the lung tissue. Histopathological analysis: removed lung tissue slices were fixed, embedded, sectioned at 3-4 *μ*m, and stained with hematoxylin and eosin; they were then observed under a microscope (100x). The inflammation score represents the severity of inflammatory cell infiltration in the airway. Representative photomicrographs from each group (*n*  =  8 per group) were shown as follows: (a) saline; (b) OVA reexposure; (c) dexamethasone (Dex) (1 mg/kg/day); (d) rapamycin (Rapa) (4 mg/kg/day); (e) AS-IV 10 mg/kg group; (f) AS-IV 20 mg/kg; and (g) AS-IV 40.0 mg/kg group. The data were expressed as the mean ± SEM. ^*∗∗*^*P* < 0.01 versus the OVA reexposure group.

To demonstrate the inhibitory effects of AS-IV on inflammation in the lung tissue, pulmonary pathology was observed by hematoxylin and eosin staining. As shown in Figure 4, a significant infiltration of inflammatory cells into peribronchiolar and perivascular connective tissues was observed in OVA reexposed mice. In contrast, AS-IV treatment remarkably attenuated airway inflammation at the dose of 40.0 mg/kg. Both rapamycin and dexamethasone treatment significantly inhibited inflammation in lung tissues.

### 3.4. AS-IV Had Limited Effects on Treg Cell Populations in BALF

**Figure 5:**
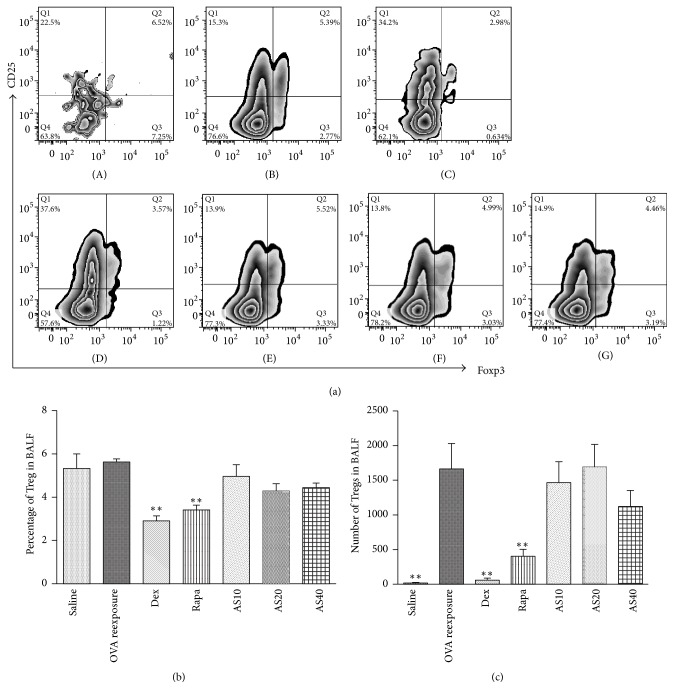
Treg cell populations in BALF. (a) Representative graphs from each group were shown as follows: (A) saline; (B) OVA reexposure; (C) dexamethasone (Dex) (1 mg/kg/day); (D) rapamycin (Rapa) (4 mg/kg/day); (E) AS-IV 10 mg/kg group; (F) AS-IV 20 mg/kg; and (G) AS-IV 40.0 mg/kg group. (b) Foxp3^+^CD25^+^ cells, as a percentage of the total BALF CD4^+^ T cells, were reduced after the administration of Rapa and Dex. However, they were not reduced by AS-IV administration (*n* = 6 mice/group). (c) Total BALF regulatory T cells (Foxp3^+^CD25^+^) were increased after OVA reexposure and suppressed by Rapa and Dex, but they were not suppressed by AS-IV. (*n* = 6 mice/group). The data were expressed as the mean ± SEM. ^*∗∗*^*P* < 0.01 versus OVA reexposure.

The mTOR signaling pathway has been demonstrated to play a significant role in the growth and proliferation of Treg cells which have an arsenal of mechanisms in asthma. Therefore, we focused on the effects of AS-IV on these cells. When regulatory T cells were assessed as a percentage of CD4^+^ T cells, rapamycin and dexamethasone obviously suppressed regulatory T cells (Figure 5(b)). Total Foxp3^+^CD25^+^ Treg cell numbers were remarkably increased in OVA reexposed mice compared to saline controls and they were completely suppressed by rapamycin and dexamethasone treatment (Figure 5(c)). However, neither the Treg cell numbers nor the percentage was significantly changed by the administration of AS-IV at 10, 20, or 40.0 mg/kg.

### 3.5. AS-IV Blocked mTORC1, but Not mTORC2 Signaling

**Figure 6:**
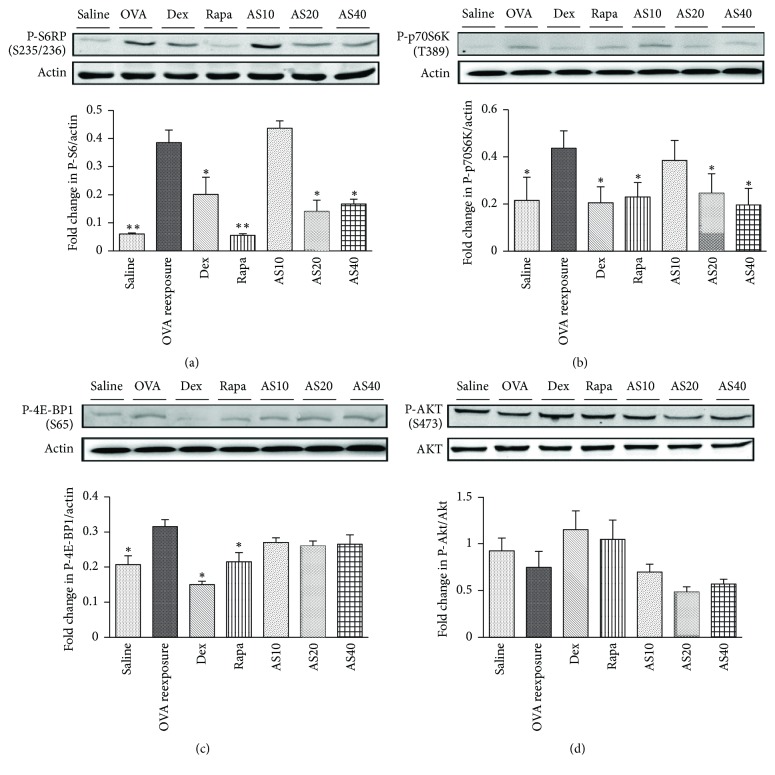
The effect of AS-IV on the mTOR pathway in lung homogenates. (a), (b), and (c) Western blot analysis for the activation status of mTORC1. P-S6 (Ser235/236) and P-p70S6K (Thr389), the downstream mediators of mTOR complex 1 signaling, were increased in OVA reexposed mice, which were blocked by AS-IV at doses of 20 and 40 mg/kg, as well as rapamycin (Rapa) (4 mg/kg/day) and dexamethasone (Dex) (1 mg/kg/day) treatment (*n* = 6 mice/group). However, increased phosphorylation of 4E-BP1 (Ser65), another substrate of mTORC1, was only inhibited by rapamycin (Rapa) (4 mg/kg/day) and dexamethasone (Dex) (1 mg/kg/day), and AS-IV (10, 20, or 40 mg/kg) had little effects on P-4E-BP1 level. (d) P-Akt, a downstream mediator of mTOR complex 2 signaling, was increased after allergen reexposure compared to mice that had 3 weeks of rest (*n* = 6 mice/group), but it was unaffected by AS-IV, Dex, or Rapa. The data are expressed as the mean ± SEM. ^*∗*^*P* < 0.05 and ^*∗∗*^*P* < 0.01 versus OVA reexposure.

mTOR has previously been shown to play an important role in the pathogenesis of asthma. To determine whether the effects of AS-IV on airway inflammation were associated with mTOR pathway, the mTORC1 and mTORC2 activities were assessed by phosphorylation of their substrates S6RP, p70S6K, 4E-BP1, and Akt, respectively. OVA reexposure was induced to a significant augmentation of the S6RP, p70S6K, and 4E-BP1 phosphorylation compared to saline controls. The enhanced phosphorylation of S6RP and p70S6K but not 4E-BP1 was significantly inhibited by AS-IV treatment at doses of 20.0 and 40.0 mg/kg, as well as rapamycin and dexamethasone treatment. However, phosphorylation of Akt was not suppressed by AS-IV, dexamethasone, or rapamycin treatment. Taken together, these findings indicated that the AS-IV could effectively block mTORC1, but not mTORC2 (Figure 6).

## 4. Discussion

It is beneficial to keep patients in asthma remission and avoid relapse.* Astragalus membranaceus*, a traditional Chinese medicinal herb, has been widely used in asthma remission to prevent an asthma attack after reexposure to an allergen. Previous studies have demonstrated that AS-IV, an important derivative of* Astragalus membranaceus,* could alleviate airway inflammation when it was administered early in asthma. However, relevant data are still scarce especially of AS-IV on established asthma models. To further understand the antiasthmatic mechanism of AS-IV in the remission stage, a unique established mouse model of asthma was used in the present study. Consistent with previous reports, OVA reexposure caused significant airway inflammatory changes, and we confirmed the inhibitory effects of AS-IV on airway inflammation and AHR. In line with the published data, AS-IV was confirmed to suppress the expression of Th2 cytokines including IL-4 and IL-5, and increased Th1 cytokine like INF-*γ*. It seems that regulation of Th1/Th2 cytokine imbalance may contribute to the antiasthmatic effects of AS-IV. Furthermore, AS-IV was found to reduce the IL-17A level in BALF of asthma mice, which might indicate that AS-IV could regulate Th17 dysfunction. Nevertheless, the pathogenesis of bronchial asthma is extremely complex, and mechanisms through which AS-IV exerts its antiasthmatic effects in established allergic disease remain unclear.

mTOR signaling occurs downstream of the PI3K-signaling cascade and is known to play a significant role in the pathogenesis of asthma [12]. mTOR is a highly conserved and ubiquitous serine-threonine Kinase including two protein complexes, mTORC1 and mTORC2. Inhibition of mTOR signaling has immunosuppressive effects on T cells, antigen-presenting cells, B cells, and NKT cells that participate in the pathogenesis of asthma [13–15]. mTORC1 inhibition by rapamycin has been shown to attenuate Th2 and Th17 inflammation in asthma models [16, 17], whereas the mTORC2/Akt pathway plays an important role in modulating airway smooth muscle remodeling in asthma [18]. AS-IV has been suggested to exert cardiac protection through inhibition of mTORC1 signaling [10]. Additionally, AS-IV ameliorates renal injury via mTOR inhibition [19]. Moreover, the* Astragalus* saponins that contain AS-IV have been shown to exhibit anticancer effects via modulating the mTOR signaling pathway [20, 21]. Based on the pivotal role of mTOR signaling in the pathogenesis of asthma, we investigated whether the antiasthmatic effects of AS-IV were associated with the mTOR signaling. The mTORC1 and mTORC2 activities were assessed by the phosphorylation of their downstream targets, S6RP, p70S6K, 4E-BP1, and Akt, respectively. In this study, AS-IV prominently suppressed OVA-induced increases in the phosphorylated p70S6K and S6RP levels, but phosphorylation of Akt was not significantly changed. These results indicated that AS-IV could effectively block mTORC1 without affecting mTORC2. Interestingly, we showed that mTORC1 inhibition by AS-IV affected S6K1/S6RP but not 4E-BP1 phosphorylation status. An explanation for this observation might be the incomplete inhibition of mTORC1 by AS-IV. However, an alternative explanation might be that S6K1 and 4E-BP1 were independently regulated, and 4E-BP1 phosphorylation has been demonstrated to be regulated directly or indirectly by PI3K but not via mTORC1 [22].

As supporting evidence for the current study, it has been suggested that inhibition of mTOR with rapamycin has widely immunosuppressive activities at their disposal to attenuate AHR and airway inflammation in experimental allergic asthma [5]. Previous studies demonstrated that Th2 polarization and function depend on the mTORC1 pathway, and rapamycin was proved to inhibit Th2 cell proliferation via a mTORC1/S6 Kinase-1-dependent pathway [23, 24]. The best characterized source of IL-17 is Th17 cells, the differentiation of which was demonstrated to be dependent on the mTORC1 pathway [4, 25]. Taken together, we suggest the decreased IL-4, IL-5, or IL-17 cytokine by rapamycin or astragaloside IV were possibly attributed to inhibition of Th2 and Th17 cells, respectively, via a mTORC1-dependent pathway. We previously found astragaloside IV could suppress Th2 response* in vitro* by inhibiting GATA-3 expression of D10.G4.1 cell (a Th2 cell line), providing another rationale that the antiasthmatic effects of astragaloside IV were attributed to the inhibition of Th2 cells. However, regardless of the cellular source of IL-4, IL-5, and IL-17 (Th2 cells, Th17 cells, mast cells, eosinophils, and/or CD8+ T cells), the observed reduction in AHR and airway inflammation by AS-IV may be a result of inhibitory effects on IL-4, IL-5, and IL-17, which are secondary to the mTORC1 inhibition. An interesting observation in our studies was that Th1 cytokine INF-*γ* was increased by AS-IV, though mTORC1 was inhibited. This was surprising since Th1 differentiation is mostly dependent on mTORC1 signaling. It was possible that AS-IV could promote INF-*γ* from Th1 cells independent of mTORC1 pathway, but further studies have to reveal the mechanism of AS-IV increasing INF-*γ* level. Of note, the antiasthmatic effects of AS-IV in this study were at least partially attributed to the inhibition of the mTORC1 activity.

Recently, a crucial role for CD4^+^CD25^+^Foxp3^+^ Treg cells in the resolution of allergic airway disease has been increasingly recognized [26, 27]. The mTOR signaling pathway plays a necessary role in the differentiation and proliferation of Treg cells, and inhibition of mTOR by rapamycin in CD4^+^ T cells induced the expansion of the naturally occurring CD4^+^CD25^+^Foxp3^+^Treg cells* in vitro* [28]. However, recent studies showed that Treg cells were differentiated only when both mTORC1 and mTORC2 were inhibited [29]. In the present study, AS-IV blocked mTORC1 signaling, but not mTORC2. In line with this observation, we found that AS-IV treatment did not significantly increase the number or proportion of CD4^+^CD25^+^Foxp3^+^ Treg cells. However, previous studies from our lab demonstrated that the number of CD4^+^CD25^+^Foxp3^+^ Treg cells was increased by* Astragalus membranaceus* from which AS-IV could be isolated [30]. It is possible that* Astragalus membranaceus* contains other components, such as* Astragalus* polysaccharide that may be mediated by enhancement of CD4^+^CD25^+^Foxp3^+^ Tregs. In line with this,* Astragalus* polysaccharide was demonstrated to possess functions of inducing regulatory T cells [31]. Moreover, the effects of AS-IV and* Astragalus membranaceus* may be different when used in different dosage. Nevertheless, further studies are still needed to illustrate their contradictory effects on Tregs in different diseases.

In the present study, the antiasthmatic effects of AS-IV were compared to those of dexamethasone and rapamycin. Similar to AS-IV, previous studies have shown that dexamethasone and rapamycin could inhibit mTOR signaling pathway [5, 32]. In line with the published data, the present study confirmed that dexamethasone and rapamycin both inhibited mTORC1 activity, which accounted for their beneficial effects on the airway inflammation, AHR, and increased Th2 cytokines in our asthma model. It suggests that rapamycin can promote the differentiation and proliferation of Treg cells in the presence of IL-2* in vitro* [28]. However, in our* in vivo* model, CD4^+^CD25^+^Foxp3^+^ Tregs were surprisingly found to be significantly reduced by rapamycin, much like dexamethasone treatment. It was possible that rapamycin and dexamethasone might decrease Treg cells* in vivo* by decreasing the number of IL-2 producing cells. Moreover, the dosage of rapamycin used in this study only inhibited mTORC1 activities, which, as illustrated above, was not enough to promote differentiation of Treg cells. Taken together, AS-IV, in comparison with dexamethasone and rapamycin, had comparable effects on the airway inflammation and AHR in our asthma model, but without reducing the Treg cells.

## 5. Conclusion

The present study confirmed that AS-IV could effectively attenuate airway inflammation in an established murine model of asthma when reexposed to an antigen. Noteworthily, the results of this study provide new insights into the antiasthmatic effects of AS-IV that are at least partially through inhibition of the mTORC1 signaling pathway.
